# NephroCheck AKI risk scores (TIMP-2 and IGFBP7) in pregnancy

**DOI:** 10.1093/ndt/gfaf031

**Published:** 2025-02-14

**Authors:** Katherine Clark, Jennifer Joslin, Carolyn Gill, Priscilla Smith, Hayley Martin, Hayley Tarft, Frances Conti-Ramsden, Lucy C Chappell, Marlies Ostermann, Kate Bramham

**Affiliations:** King's College Hospital NHS Foundation Trust, London, UK; Department of Women and Children's Health, School of Life Course and Population Sciences, Faculty of Life Course and Population Sciences, King's College London, London, UK; King's College Hospital NHS Foundation Trust, London, UK; Department of Women and Children's Health, School of Life Course and Population Sciences, Faculty of Life Course and Population Sciences, King's College London, London, UK; Department of Women and Children's Health, School of Life Course and Population Sciences, Faculty of Life Course and Population Sciences, King's College London, London, UK; King's College Hospital NHS Foundation Trust, London, UK; Department of Women and Children's Health, School of Life Course and Population Sciences, Faculty of Life Course and Population Sciences, King's College London, London, UK; King's College Hospital NHS Foundation Trust, London, UK; Guy's and St Thomas’ NHS Foundation Trust, London, UK; Department of Women and Children's Health, School of Life Course and Population Sciences, Faculty of Life Course and Population Sciences, King's College London, London, UK; Department of Women and Children's Health, School of Life Course and Population Sciences, Faculty of Life Course and Population Sciences, King's College London, London, UK; Guy's and St Thomas’ NHS Foundation Trust, London, UK; Guy's and St Thomas’ NHS Foundation Trust, London, UK; King's College Hospital NHS Foundation Trust, London, UK; Department of Women and Children's Health, School of Life Course and Population Sciences, Faculty of Life Course and Population Sciences, King's College London, London, UK

To the Editor,

Pregnancy-associated acute kidney injury (PrAKI) is associated with increased morbidity and mortality for both the woman and the baby compared with those without PrAKI [1]. Even after complete recovery from any previous AKI events, future pregnancies may be adversely affected [2].

There are currently no validated diagnostic markers for PrAKI. Due to dynamic changes in serum creatinine (SCr) concentration across gestation [3, 4], equations used to estimate the glomerular filtration rate (GFR) are inaccurate in pregnancy [5] and adaptation of pre-existing AKI diagnostic algorithms using SCr is likely needed. Diagnosis based on urine output is not reliable, as oliguria may occur physiologically during the peripartum period [6] in response to interventions such as oxytocin administration and in the absence of a bladder catheter. The lack of standardized intrapartum fluid management protocols adds further uncertainty, thus urine output is not used as an independent diagnostic factor for PrAKI.

A promising AKI predictor in non-pregnant patients is the NephroCheck AKI Risk Score. It is derived from the product of two urinary G1 cell cycle arrest markers: tissue inhibitor of metalloproteinase-2 (TIMP-2) and insulin-like growth factor binding protein 7 (IGFBP7), which indicate tubular cell stress before a SCr increase occurs [7]. Numerous studies in critically ill patients have demonstrated an incremental risk of AKI (stages 2 and 3) with a urinary [TIMP-2] × [IGFBP7] >0.3(ng/ml)^2^/10^3^ [7, 8], but its association with PrAKI has not been assessed. To determine if measurement of [TIMP-2] × [IGFBP7] could facilitate early PrAKI diagnosis and guide prevention strategies in women with and without risk factors, we examined whether urinary [TIMP-2] × [IGFBP7] concentrations using described medium- and high-risk AKI adult (non-pregnant) thresholds would be applicable to a pregnant cohort.

We performed two studies at two tertiary centres in the UK (2017, 2021). The first study aimed to determine the role of urinary [TIMP-2] × [IGFBP7] measurement in women with suspected pre-eclampsia to detect PrAKI; the second aimed to compare [TIMP-2] × [IGFBP7] concentrations in pregnant and non-pregnant healthy women without AKI. Urinary [TIMP-2] × [IGFBP7] concentrations were determined with the handheld Astute meter (Astute Medical, San Diego, CA, USA) [9] using mid-stream urine samples according to the manufacturer's instructions. AKI within 7 days of testing was defined according to Kidney Disease: Improving Global Outcomes (KDIGO) SCr criteria [10]. Pre-eclampsia was diagnosed according to the 2014 International Society for the Study of Hypertension in Pregnancy definition [11] utilized at the time of the study (subsequent updates include new criteria that are less relevant to PrAKI diagnosis [12]).

A total of 116 women who were seen in the maternal assessment unit with suspected pre-eclampsia (systolic blood pressure >140 mmHg or diastolic blood pressure >90 mmHg and/or new-onset proteinuria ≥2+ on urinalysis), recruited to the Preeclampsia, Chronic Hypertension, rEnal and SLE (PEACHES) study were included (REC 11/LO/1776) (Table 1, Study 1). All had a body mass index <30 kg/m^2^ and no diabetes. Urine samples were taken for analysis and usual care pathways were followed. A total of 65/116 (56.0%) women had a diagnosis of pre-eclampsia that was subsequently confirmed, of which 4/65 (6.2%) also had PrAKI based on the KDIGO SCr criteria. A total of 4/116 (3.4%) women had PrAKI without pre-eclampsia. All PrAKI patients in this cohort were stage 1, with a peak median SCr of 114 µmol/l [interquartile range (IQR) 95–126].

In this cohort with suspected pre-eclampsia, 57/116 (49.1%) women had urinary [TIMP-2] × [IGFBP7] >0.3(ng/ml)^2^/10^3^, of which 23/57 (40.4%) women had no subsequent diagnosis of PrAKI or pre-eclampsia. There was no relationship between [TIMP-2] × [IGFBP7] and gestation, urinary protein:creatinine ratio (UPCR) at the time of urine sampling or the highest subsequent SCr concentration during the pregnancy. There was also no correlation between [TIMP-2] × [IGFBP7] and SCr concentration at the time of sampling (see Supplementary Information). There was no significant difference in urinary [TIMP-2] × [IGFBP7] results between women with subsequent confirmed pre-eclampsia [median 0.30(ng/ml)^2^/10^3^ (IQR 0.15–0.72)] and those without [median 0.25(ng/ml)^2^/10^3^ (IQR 0.12–0.60)], even after exclusion of those with PrAKI. This supports findings from a small study that found

**Table 1:** Demographics and AKI risk scores for all groups sampled across both studies.

	Study 1: Urinary [TIMP-2] × [IGFBP7] in women recruited with suspected pre-eclampsia (*n* = 116)				Study 2: Urinary [TIMP-2] × [IGFBP7] in healthy pregnant women versus non-pregnant controls (*n* = 40)	
Characteristics	No confirmed pre-eclampsia or AKI (*n* = 47)	Pre-eclampsia, no PrAKI (*n* = 61)	Pre-eclampsia and PrAKI (*n* = 4)	PrAKI, no pre-eclampsia (*n* = 4)	Non-pregnant (*n* = 20)	Healthy pregnant (*n* = 20)
Age (years), median (IQR)	31.7 (27.6–35.9)	32.6 (27.8–34.7)	30.2 (24.8–37.2)	29.7 (21.4–30.7)	34.5 (27.0–36.5)	33.3 (31.3–35.6)
Ethnicity, *n* (%)						
White	19 (40.4)	14 (22.9)	0 (0.0)	0 (0.0)	13 (65.0)	15 (75.0)
Black	12 (25.5)	22 (36.1)	1 (25.0)	2 (50.0)	1 (5.0)	1 (5.0)
Asian	5 (10.6)	5 (8.2)	1 (25.0)	0 (0.0)	2 (10.0)	1 (5.0)
Other	2 (4.3)	6 (9.8)	1 (25.0)	1 (25.0)	4 (20.0)	3 (15.0)
Unknown	9 (19.2)	14 (22.9)	1 (25.0)	1 (25.0)	0 (0.0)	0 (0.0)
Gestation at sample (weeks), median (IQR)	34.6 (33.6–36.4)	34.6 (33.6–36.3)	37.3 (28.6–37.3)	38.3 (33.3–39.4)	N/A	35.8 (35.4–36.3)
Urinary [TIMP-2] × [IGFBP7] in (ng/ml)^2^/10^3^, median (IQR)	0.27 (0.12–0.61)	0. 27 (0.15–0.70)	0.99 (0.25–2.66)	0.30 (0.23–0.52)	0.17 (0.06–0.35)	0.28 (0.13–0.64)
Range	0.03–3.00	0.06–8.44	0.49–3.64	0.22–0.66	0.03–1.99	0.07–1.14
≤0.3, *n* (%)	24 (51.1)	32 (52.5)	1 (25.0)	2 (50.0)	13 (65.0)	10 (50.0)
0.3–2.0, *n* (%)	22 (46.8)	25 (41.0)	2 (50.0)	2 (50.0)	7 (35.0)	10 (50.0)
>2.0, *n* (%)	1 (2.1)	4 (6.5)	1 (25.0)	0 (0.0)	0 (0.0)	0 (0.0)

Pre-eclampsia per ISHHP definition [11].

no significant difference in urinary TIMP-2 or IGFBP7 values between hypertensive and control groups within 24 hours of delivery [13]. Similarly, urinary [TIMP-2] × [IGFBP7] was not associated with surrogates of pre-eclampsia severity (highest systolic blood pressure, foetal birthweight or gestation at delivery). Many pregnant women had increased urinary [TIMP-2] × [IGFBP7] concentrations without developing KDIGO-defined AKI, regardless of pre-eclampsia diagnosis, SCr or gestation at sampling, highest blood pressure or UPCR. There was no relationship between ethnicity and urinary [TIMP-2] × [IGFBP7] concentration, although women of White ethnicity were significantly less likely to be diagnosed with PrAKI or pre-eclampsia (*P* < .001).

We also explored if different thresholds of urinary [TIMP-2] × [IGFBP7] may be required for prediction of PrAKI compared with outside of pregnancy (Study 2). Therefore, we determined urinary [TIMP-2] × [IGFBP7] concentrations in 20 healthy pregnant women without evidence of complications (including PrAKI, hypertension, renal disease, diabetes, BMI >30 kg/m^2^ and other known causes and risk factors of PrAKI) and a median gestation of 35.8 weeks (IQR 35.4–36.3) and 20 non-pregnant controls (Table 1, Study 2).

**Figure 1:**
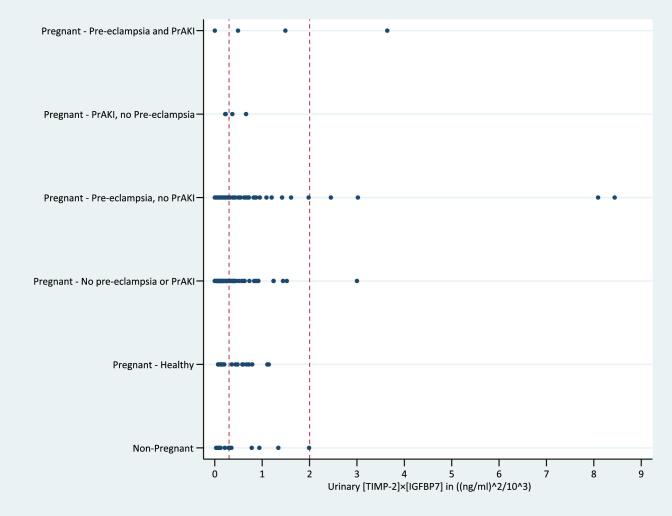
Scatter plot of AKI risk scores by group with reference lines at 0.3 and 2.0 (ng/ml)^2^/10^3^.

The median urinary [TIMP-2] × [IGFBP7] results in healthy pregnant women tended to be higher than in non-pregnant women, but this result was not significantly different [0.28(ng/ml)^2^/10^3^ (IQR 0.07–1.14) versus 0.17(ng/ml)^2^/10^3^ (IQR 0.03–1.99); *P* = .16]. There are minimal published data describing [TIMP-2] × [IGFBP7] results in healthy, non-pregnant cohorts, but the healthy non-pregnant cohort urinary [TIMP-2] × [IGFBP7] results in our study are similar to cohorts who have returned to health [14]. Half (10/20) of healthy pregnant women had an urinary [TIMP-2] × [IGFBP7] result >0.3(ng/ml)^2^/10^3^ compared with 35% (7/20) of healthy, non-pregnant women (Fig. 1). In total, across both studies, urinary [TIMP-2] × [IGFBP7] >2.0(ng/ml)^2^/10^3^ was observed in six pregnant women in Study 1, of which only one woman had PrAKI, one had pre-eclampsia and four had no pregnancy complications.

Urinary [TIMP-2] × [IGFBP7] results were not significantly higher in pregnant women, but the study is likely underpowered to show a difference. The high proportion of healthy pregnant women with urinary [TIMP-2] × [IGFBP7] >0.3 ng/ml^2^/10^3^, which would suggest a high risk of development of AKI stage 2 or 3 in non-pregnant individuals, indicates that a higher threshold for prediction of AKI using urinary [TIMP-2] × [IGFBP7] may be needed in pregnancy. As our study did not include any stage 2 or 3 PrAKI cases, further work investigating these groups would be beneficial.

A further challenge in the utility of measuring urinary [TIMP-2] × [IGFBP7] for the prediction of PrAKI is timing. TIMP-2 and IGFBP7 are involved in G1 cell cycle arrest and urinary levels rise 4 hours after tubular injury and peak 12 hours after, but they also normalize rapidly in the absence of persistent insult. The most common causes of PrAKI are infection, pre-eclampsia and haemorrhage [15]. Therefore, the exact timing of the renal insult may be difficult to determine compared with cohorts that have demonstrated that measurement of cell cycle arrest markers was effective and impacted clinical management (e.g. after major surgery) [16]. Indeed, in a cohort of critically ill pregnant and postpartum women there was no significant difference in [TIMP-2] × [IGFBP7] between those with and without AKI: median [TIMP-2] × [IGFBP7] was 9.1 (IQR 6.8–11.6) [17]. This suggests that an elevated score may be present in all critically ill peripartum patients and emphasizes the need for appropriate timing of testing.

Our work demonstrates that the role of a single measurement of NephroCheck in the prediction and diagnosis of PrAKI is currently limited. Assessment of the role of testing in pregnancy and peripartum scenarios more aligned with current out-of-pregnancy usage would be beneficial alongside exploration of other potential biomarkers. Further work is needed to determine the optimal timing of NephroCheck sampling in specific cohorts, the role of serial measurement and if the threshold value needs to be modified to improve accuracy in PrAKI.
